# Population-Matched Transcriptome Prediction Increases TWAS Discovery and Replication Rate

**DOI:** 10.1016/j.isci.2020.101850

**Published:** 2020-11-23

**Authors:** Elyse Geoffroy, Isabelle Gregga, Heather E. Wheeler

**Affiliations:** 1 Program in Bioinformatics, Loyola University Chicago, Chicago, IL 60660, USA; 2 Department of Biology, Loyola University Chicago, Chicago, IL 60660, USA

**Keywords:** Population, Genetics, Genomics, Human Genetics

## Abstract

Most genome-wide association studies (GWAS) and transcriptome-wide association studies (TWAS) focus on European populations; however, these results cannot always be accurately applied to non-European populations due to genetic architecture differences. Using GWAS summary statistics in the Population Architecture using Genomics and Epidemiology study, which comprises ∼50,000 Hispanic/Latinos, African Americans, Asians, Native Hawaiians, and Native Americans, we perform TWAS to determine gene-trait associations. We compared results using three transcriptome prediction models derived from Multi-Ethnic Study of Atherosclerosis populations: the African American and Hispanic/Latino (AFHI) model, the European (EUR) model, and the African American, Hispanic/Latino, and European (ALL) model. We identified 240 unique significant trait-associated genes. We found more significant, colocalized genes that replicate in larger cohorts when applying the AFHI model than the EUR or ALL model. Thus, TWAS with population-matched transcriptome models have more power for discovery and replication, demonstrating the need for more transcriptome studies in diverse populations.

## Highlights

• TWAS mechanistically extends GWAS findings in diverse populations
• Population-matched transcriptome models detect more replicable associations
• Colocalization shows GWAS variants likely act through gene expression regulation
• More GWAS and transcriptome modeling in diverse populations are needed

## Graphical Abstract

**Figure:**
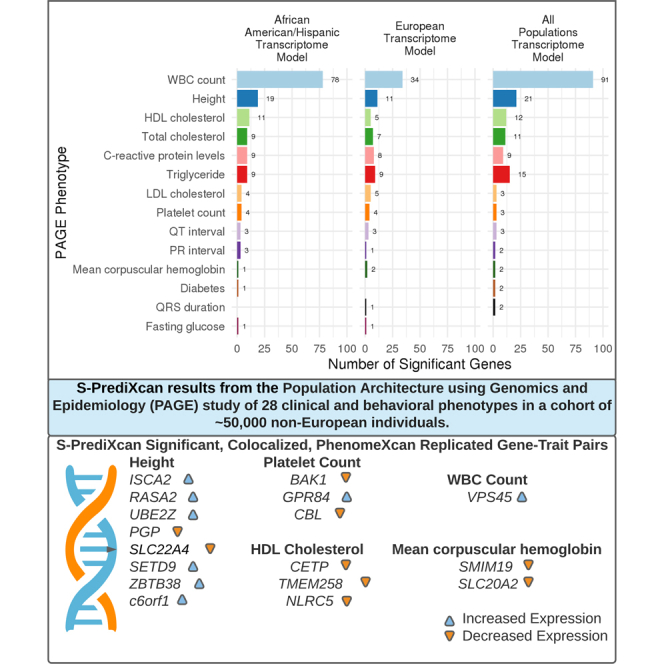

## Introduction

Genome-wide association studies (GWAS) test single-nucleotide polymorphisms (SNPs) across the genome for association with diseases and other complex traits. GWAS have identified thousands of SNP-trait associations with complex traits; however, the majority of the studies exclusively include individuals of European ancestries (Buniello *et al.*, 2019). As of 2017, within 4655 GWAS, 78% of individuals come from European ancestries (Morales *et al.*, 2018), creating a significant gap of knowledge for those of non-European descent. Even when present in large scale biobanks, non-European populations are often excluded from genetic analyses (Peterson *et al.*, 2019; Ben-Eghan *et al.*, 2020), which further worsens under-representation of diverse populations in research. As those of European ancestries only make up a small fraction of the human population, expanding the number of non-European individuals in genomic research benefits all populations by more fully incorporating global genetic diversity in association studies. Since populations were isolated from each other by geography throughout large spans of human history, allele frequencies and effect sizes differ across populations, making current GWAS results poor genetic predictors for non-European populations (Mogil *et al.*, 2018; Martin *et al.*, 2019; Keys *et al.*, 2020). To start to address this problem, the Population Architecture using Genomics and Epidemiology (PAGE) study performed 28 GWAS on clinical and behavioral phenotypes in a multi-ancestries cohort that included Hispanic/Latinos, African Americans, Asians, Native Hawaiians, and Native Americans (Wojcik *et al.*, 2019). The PAGE study is the largest collection of GWAS conducted in non-Europeans.

Meanwhile, transcriptome-wide association studies (TWAS) incorporate transcriptome data along with genotype and phenotype data to make gene-trait associations (Gamazon *et al.*, 2015; Gusev *et al.*, 2016). In TWAS, expression quantitative trait loci (eQTL) data are used to build models that predict gene expression levels from genotypes. The models are integrated with GWAS data to test genes, rather than SNPs, for association with complex traits. Gene-trait associations identified through TWAS provide evidence that gene regulatory mechanisms underlie the trait's biology. TWAS have not yet been applied to the PAGE GWAS results.

Here, we perform TWAS with S-PrediXcan (Barbeira *et al.*, 2018) in PAGE using GWAS summary statistics and three transcriptome prediction models built in the Multi-Ethnic Study of Atherosclerosis (MESA) (Bild et al., 2002, Liu et al., 2013, Mogil et al., 2018). We compared performance and replication of each transcriptome prediction model to determine whether population ancestry matching or sample size is more important in TWAS. We use one transcriptome model built in the MESA African American and Hispanic/Latino (AFHI) populations, one built in the MESA European population (EUR), and another built in the MESA African American, Hispanic/Latino, and European (ALL) populations combined. From there, we colocalize our S-PrediXcan results using COLOC software (Giambartolomei *et al.*, 2014; Hormozdiari *et al.*, 2016; Barbeira *et al.*, 2018; Pividori *et al.*, 2020; Barbeira et al., 2019) to provide more evidence the SNPs in discovered genes are acting through gene expression regulation to affect the associated phenotypes. We then tested discovered associations for replication using the PhenomeXcan database, which includes S-PrediXcan results from large, predominantly European GWAS (Pividori *et al.*, 2020). We find a higher proportion of gene-trait pairs identified in PAGE replicate when we use the population-matched AFHI transcriptome prediction model than either the EUR or ALL transcriptome prediction models. All scripts used for analyses are available at https://github.com/WheelerLab/MESA_expression_prediction.

## Results

We sought to perform TWAS in the PAGE study (Wojcik *et al.*, 2019) to reveal new associations or show that previously discovered GWAS loci likely act through transcription regulation to affect the trait. We also sought to compare TWAS results in the diverse PAGE cohort using two different transcriptome prediction models, one built in populations that more closely match the genetic ancestries of PAGE and one that is composed of individuals of European genetic ancestries. In addition, we compared these results to a third transcriptome model that included all available populations. In the PAGE study, 28 GWAS on clinical and behavioral phenotypes (Table 1) were performed (Wojcik *et al.*, 2019). Individuals in PAGE self-identified as Hispanic/Latino (n = 22,216), African American (n = 17,299), Asian (n = 4,680), Native Hawaiian (n = 3,940), Native American (n = 652), or Other (n = 1,052) (Wojcik *et al.*, 2019). In comparison to any other GWAS, this study includes the most phenotypes tested in a single study, the most trait associations, and the highest number of non-European individuals (Wojcik *et al.*, 2019). TWAS integrate genetically regulated gene expression into complex trait mapping studies, but like GWAS, most are performed in European populations (Gamazon *et al.*, 2015; Gusev *et al.*, 2016). We compared S-PrediXcan results using transcriptome prediction models trained with genotype and monocyte gene expression data from three populations in MESA to find genes associated with traits in PAGE. Two MESA models (Mogil *et al.*, 2018) were built in populations of similar size: EUR (n = 578), which comprises individuals of European ancestries and reflects transcriptome data more readily available, and AFHI (n = 585), which comprises individuals of African American and Hispanic/Latino ancestries and more closely resembles the ancestries of individuals in PAGE. However, we also use ALL (n = 1,163), which includes both EUR and AFHI individuals, to see if increased sample size with increased population diversity improves our ability to discover and replicate TWAS associations.

**Table 1:** Population Architecture Using Genomics and Epidemiology (PAGE) Phenotypes Tested in TWAS and the Significant Gene Counts for Each Phenotype and Transcriptome Prediction Model

Trait	Total N or N Cases/N Controls	Mean or % Cases	SD of Mean	TWAS with AFHI Count	TWAS with EUR Count	TWAS with all Count
Inflammatory traits						
C-reactive protein (CRP) (mg/L)	28,520	4.114	4.836	9	8	9
White blood cell (WBC) count (10^9^ cells/L)	28,608	6.253	1.943	78	34	91
Mean corpuscular hemoglobin concentration (MCHC) (g/dL)	19,803	32.909	1.249	1	2	2
Platelets (per mcL)	29,328	246.783	64.273	4	4	3
Lipid traits						
HDL cholesterol (mg/dL)^a^	33,063	50.738	15.372	11	5	12
LDL cholesterol (mg/dL)^a^	32,221	137.777	40.945	4	5	3
Triglycerides (mg/dL)^a^	33,096	137.830	92.125	9	9	15
Total Cholesterol (mg/dL)^a^	33,185	214.864	46.452	9	7	11
Lifestyle traits						
Cigarettes/day exclude nonsmokers	15,862	12.507	9.088	0	0	0
Coffee (cups/day)	35,902	0.893	1.130	0	0	0
Glycemic traits						
HbA1c (mmol/mol)^b^	11,178	36.823	4.520	0	0	0
Fasting insulin (pmol/L)^b^	21,551	10.233	7.979	0	0	0
Fasting glucose (mmol/L)^b^	23,911	5.050	0.633	1	1	0
Type 2 diabetes (cases/controls)	14,042/31,683	30.7%		1	0	2
Electrocardiogram traits						
QT interval (ms)	17,348	410.678	30.580	3	3	3
QRS interval (ms)	17,046	89.023	9.596	0	1	2
PR interval (ms)	17,422	158.909	22.364	3	1	2
Blood Pressure traits						
Systolic blood pressure (mm Hg)^a^	35,433	132.150	22.243	0	0	0
Diastolic blood pressure (mm Hg)^a^	35,433	80.681	13.827	0	0	0
Hypertension (cases/controls)	27,123/22,018	55.2%		0	0	0
Anthropometric traits						
WHR-females^b^	24,838	0.855	0.082	0	0	0
WHR-males^b^	9,066	0.952	0.066	0	0	0
WHR	33,904	NA	NA	0	0	0
Height (cm)	49,796	163.893	9.568	19	11	21
BMI (kg/m^2^)	49,335	29.333	6.285	0	0	0
Kidney traits						
Chronic kidney disease (cases/controls)	4,154/41,573	10.0%		0	0	0
End-stage renal disease (cases/controls)	602/32,459	1.9%		0	0	0
eGFR (mL/min)^c^	27,900	90.548	21.880	0	0	0

Phenotype information and GWAS sample sizes were taken from Table S1 in Wojcik et al., 2019. Wojcik et al., 2019 had a combined Nmax = 49,839.

SD = standard deviation; WHR = waist-to-hip ratio; HbA1c = hemoglobin A1c; eGFR = estimated glomerular filtration rate; CRP = c-reactive protein; MCHC = mean corpuscular hemoglobin concentration; BMI = body mass index; AFHI = African American and Hispanic/Latino transcriptome prediction model; EUR = European transcriptome model; ALL = African American, Hispanic/Latino, and European transcriptome model; MESA = Multi-Ethnic Study of Atherosclerosis; PAGE = Population Architecture using Genomics and Epidemiology study.

a Traits have been adjusted for medications by adding a constant.

b Traits have been adjusted for BMI.

c Estimated glomerular filtration rate (eGFR) was calculated using the CKD-EPI (Chronic Kidney Disease Epidemiology Collaboration) formula from Levey et al., 2009. See Wojcik et al., 2019 for details.

### TWAS Identifies More Significant Genes when Using Larger and Population-Matched Gene Expression Prediction Models

**Figure 1:**
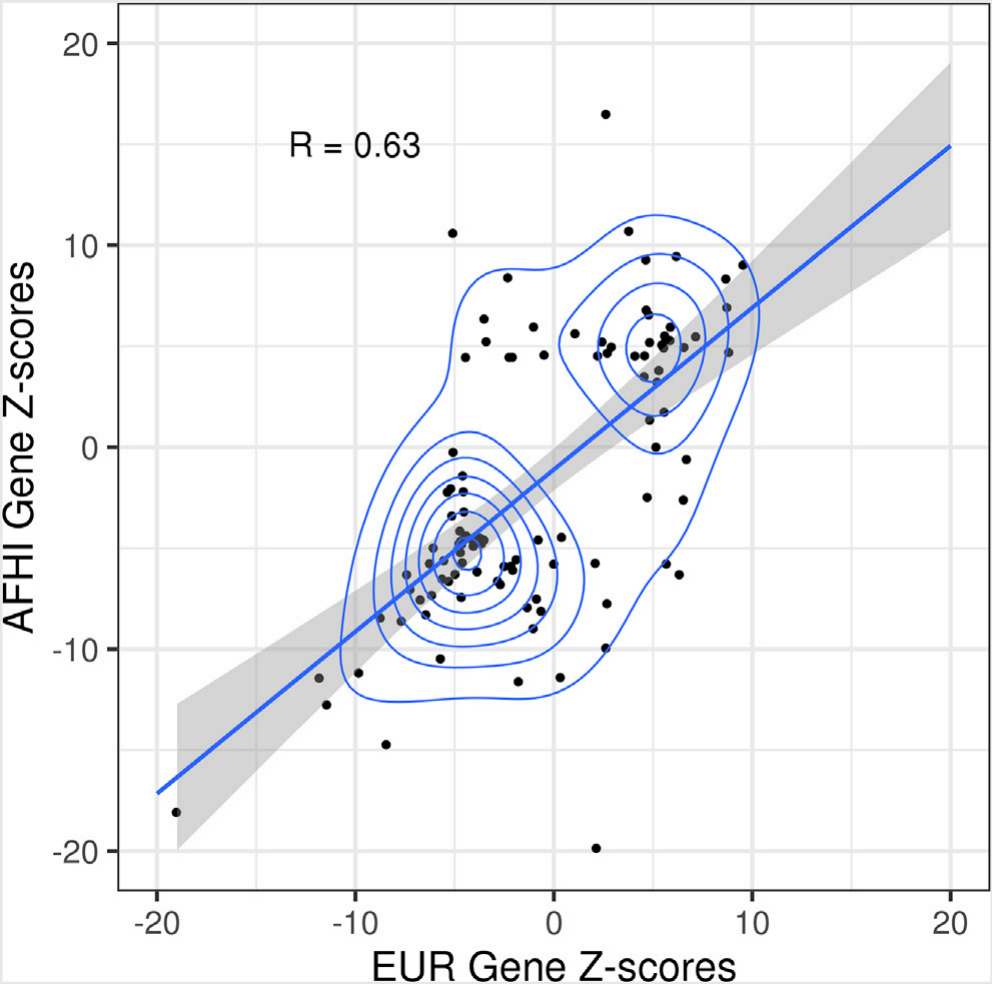
*Z* score Comparison of TWAS Significant Genes Identified by AFHI and EUR MESA Transcriptome Prediction Models in PAGE Gene-trait pairs that were identified as significant (P < 0.05/n, n = the number of genes in the transcriptome model tested in S-PrediXcan) by either model are displayed. The Pearson correlation of displayed gene-trait pairs is shown in the upper left corner (R = 0.63). AFHI = African American and Hispanic/Latino transcriptome prediction model; EUR = European transcriptome prediction model; MESA = Multi-Ethnic Study of Atherosclerosis; PAGE = Population Architecture using Genomics and Epidemiology study.

**Figure 2:**
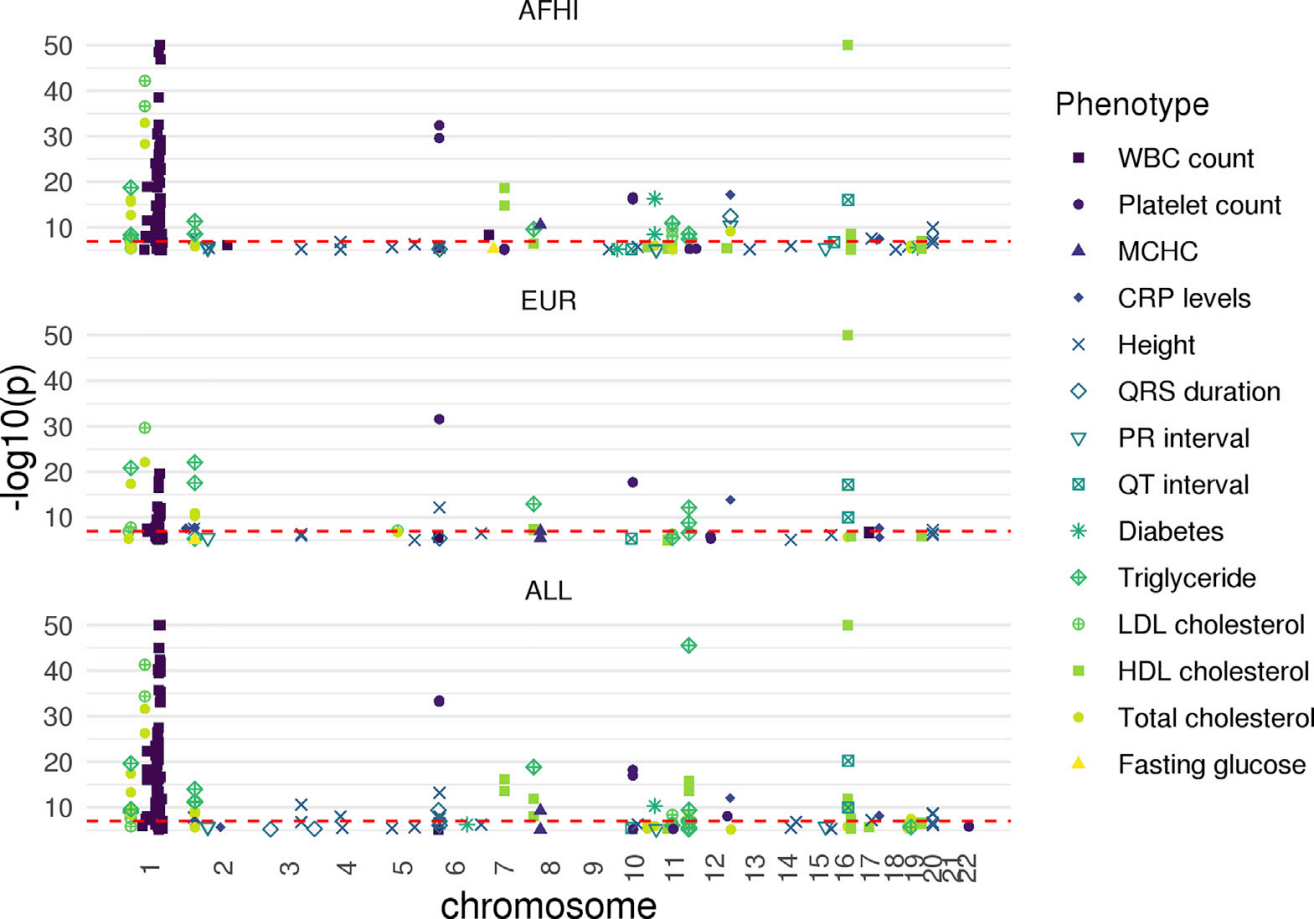
Manhattan Plot of the 14 of 28 PAGE Phenotypes Tested that Returned Significant TWAS Gene-Trait Pairs Using the AFHI, EUR, and ALL MESA Gene Expression Prediction Models Each point represents the -log10(p) of a gene association test and gene chromosomal position colored by phenotype. Only significant gene-trait pairs are shown (P < 0.05/n, n = the number of genes in the transcriptome model tested in S-PrediXcan). The dotted line is at the more conservative significance threshold calculated using all tests (P < 1.1 × 10^−7^). 11 phenotypes have gene associations that meet this more stringent threshold. Using the AFHI, EUR, and ALL models, we identified 95, 46, and 121 significant gene-trait pairs, respectively, at this threshold. Gene-trait pairs with P < 1e-50 are displayed at P = 1e-50 for readability. AFHI = African American and Hispanic/Latino transcriptome prediction model; EUR = European transcriptome model; ALL = African American, Hispanic/Latino, and European transcriptome model; MCHC = mean corpuscular hemoglobin concentration; CRP levels = c-reactive protein levels; WBC count = white blood cell count; MESA = Multi-Ethnic Study of Atherosclerosis; PAGE = Population Architecture using Genomics and Epidemiology study.

We used S-PrediXcan with the summary statistics from the 28 PAGE GWAS and either the AFHI, EUR, or ALL MESA transcriptome prediction models to perform TWAS. We found 14 of the 28 different PAGE phenotypes returned significant gene-trait associations (Table 1). We identified 152 significant gene-trait pairs with the AFHI transcriptome prediction model, 91 significant gene-trait pairs with the EUR transcriptome prediction model, and 176 significant gene-trait pairs with the ALL transcriptome prediction model (Table S1, P < 0.05/n, where n is the number of genes tested for association with each trait). In total, we identified 206 unique genes and 240 unique gene-trait pairs. Of the 240 unique gene-trait pairs, we found 50 using all three MESA models, 53 using both AFHI and EUR MESA models, 63 using AFHI and ALL MESA models, 13 using EUR and ALL MESA models, and 57 overlapped with gene-trait pairs previously mapped as a nearby gene to SNPs discovered in the original PAGE GWAS (Table S1) (Wojcik *et al.*, 2019). The Z-scores of the AFHI and EUR identified genes are highly correlated (R = 0.63), indicating that most genes have similar effects across population models and just miss reaching the significance threshold in one population or the other (Figure 1). This *Z* score correlation remains when all tested genes, not just those that reached significance with one population model, are compared (R = 0.69, Figure S1). If we are more conservative in our TWAS multiple testing adjustment and correct for all tests performed, not just tests within a trait, 95 gene-trait pairs remain significant with AFHI, 46 gene-trait pairs with EUR, and 121 gene-trait pairs with ALL (P < 1.1 × 10^−7^, Figure 2, Table S1).

### Colocalization of TWAS Results Identifies SNPs Most Likely to Act through Gene Expression Regulation

Across all TWAS phenotypes, white blood cell (WBC) count had the highest number of significant genes for each transcriptome model. We identified 34 genes (91% on chromosome 1) significantly associated with WBC count using EUR models, 78 genes (96% on chromosome 1) using AFHI models, and 91 genes (99% on chromosome 1) using ALL models. Because linkage disequilibrium and gene co-regulation are potential confounders of TWAS results (Giambartolomei *et al.*, 2014; Hormozdiari *et al.*, 2016; Barbeira *et al.*, 2018; Pividori *et al.*, 2020; Gamazon *et al.*, 2015; Wainberg *et al.*, 2019), we further investigated whether the TWAS gene associations had colocalized signals with known eQTLs. Colocalization provides additional evidence that the SNPs in a given expression model are functioning via gene expression regulation to affect the associated trait (Giambartolomei *et al.*, 2014; Hormozdiari *et al.*, 2016; Barbeira *et al.*, 2018; Pividori *et al.*, 2020).

We applied COLOC (Giambartolomei *et al.*, 2014) with the PAGE GWAS summary statistics and the AFHI, EUR, and ALL MESA eQTL data (Mogil *et al.*, 2018). Only the SNPs that were included in the MESA model and the GWAS summary statistics were tested. This allows us to determine if eQTLs are shared between the gene expression prediction models and the GWAS results. In our S-PrediXcan analyses, we identified 152, 91, and 176 genome-wide significant gene-trait pairs using the AFHI, EUR, and ALL models, respectively. Of these gene-trait pairs, 32 AFHI gene-trait pairs, 20 EUR gene-trait pairs, and 37 ALL gene-trait pairs had a colocalization probability P4 > 0.5, suggesting the eQTL and GWAS signals are colocalized. Six of the gene-trait pairs were significant in all three (AFHI, EUR, and ALL) analyses. 13 gene-trait pairs were significant in only the AFHI and ALL analyses while another three gene-trait pairs were significant in the EUR and ALL analyses. 228 gene-trait pairs between AFHI, EUR, and ALL (70, 60, and 98 gene-trait pairs, respectively) were found to be independent (P3 > 0.5). However, COLOC could not confirm 50, 11, and 41 gene-trait pairs as either colocalized or independent signals (P3 < 0.5 and P4 < 0.5) in the AFHI, EUR, and ALL models, respectively. Whether these genes are contributing to their respective traits through gene expression regulation is unknown with current data and colocalization models.

### More AFHI-Discovered Gene-Trait Pairs Replicate in PhenomeXcan Than EUR- or ALL-Discovered Gene-Trait Pairs

To determine if the gene associations we identified in PAGE replicated in TWAS studies of larger European populations, we used PhenomeXcan, a gene-trait association resource (Pividori *et al.*, 2020). PhenomeXcan is a gene-based resource with the S-MultiXcan cross-tissue gene-trait association results from UK BioBank GWAS Summary Statistics, other accessible large-scale GWAS, and the Genotype-Tissue Expression Project (GTEx) version 8 models (Pividori *et al.*, 2020; GTEx Consortium, 2020).

We tested the 62 unique colocalized gene-trait pairs for replication in the PhenomeXcan database, which includes results from larger European TWAS. We considered PhenomeXcan genes with P < 0.0008 (Bonferroni correction for 62 tests) and the same direction of effect with the same or similar trait as the discovery in PAGE to have replicated. Of the 32 AFHI colocalized discoveries, 11 (0.34) replicated in PhenomeXcan, of the 20 EUR discoveries, 5 (0.25) replicated in PhenomeXcan, and of the 37 ALL colocalized discoveries, 10 (0.27) replicated in PhenomeXcan with the same direction of effect (P < 0.0008 Table S2). Two of the PhenomeXcan replicated gene-trait pairs, *BAK1* with platelet count and *SLC22A4* with height, were significant in the AFHI, EUR, and ALL TWAS.

PhenomeXcan also reports the FASTENLOC calculated regional colocalization probabilities (RCPs) that are greater than 0.1. Given the conservative nature of colocalization approaches, this threshold limits reporting of false negatives (Pividori *et al.*, 2020). When looking at the gene-trait pairs that replicated in PhenomeXcan, all gene-trait pairs had at least one study with an RCP >0.5, which provides strong evidence that these genes are colocalized and contributing to the trait through gene expression regulation (Table 2). These genes are *ZBTB38, SLC22A4, SLC20A2, SMIM19, SETD9, CBL,* and *BAK1.*

**Table 2:** S-PrediXcan Significant Genes in PAGE with Colocalization Probability (P4) > 0.5 that Replicated in Independent Studies in PhenomeXcan

Gene Name	Z Score	Effect Size	P	CHR	P3	P4	Model	Phenotype	Best PhenomeXcan P	RCP
*CETP*	−18	−12	4.2 × 10^−73^	16	2.3 × 10^−3^	1	AFHI	HDL cholesterol	6.1 × 10^−97^	NA
*TMEM258*	−4.8	−17	1.7 × 10^−6^	11	7.1 × 10^−3^	0.95	AFHI	HDL cholesterol	1.6 × 10^−6^	NA
*SETD9*	4.7	−9.7	2.3 × 10^−6^	5	0.19	0.80	AFHI	Height	9.6 × 10^−17^	0.57
*RASA2*	4.5	−7.7	5.7 × 10^−6^	3	6.5 × 10^−2^	0.92	AFHI	Height	2.1 × 10^−105^	NA
*UBE2Z*	5.4	9.4	2.7 × 10^−8^	17	0.23	0.77	AFHI	Height	4.5 × 10^−48^	NA
*ISCA2*	4.8	0.09	1.3 × 10^−6^	14	0.03	0.97	AFHI	Height	5.8 × 10^−25^	NA
*SLC22A4*	−5.0	−0.05	5.3 × 10^−7^	5	0.17	0.81	AFHI	Height	6.2 × 10^−47^	NA
*SMIM19*	−6.6	0.16	3.1 × 10^−11^	8	0.10	0.90	AFHI	MCHC	2.8 × 10^−23^	0.58
*BAK1*	−11	0.02	2.6E-30	6	4.4 × 10^−3^	1	AFHI	Platelet count	2.6 × 10^−149^	0.97
*CBL*	−4.5	−0.06	6.0 × 10^−6^	11	1.8 × 10^−2^	0.98	AFHI	Platelet count	6.9 × 10^−60^	0.81
*VPS45*	9.7	−0.05	3.9 × 10^−22^	1	2.2 × 10^−2^	0.95	AFHI	WBC count	5.8 × 10^−6^	NA
*ZBTB38*	4.9	−0.11	1.2 × 10^−6^	3	1.7 × 10^−2^	0.98	EUR	Height	9.5 × 10^−150^	0.58
*PGP*	−4.9	−2.6	8.0 × 10^−7^	16	6.7 × 10^−3^	0.99	EUR	Height	1.9 × 10^−32^	NA
*SLC22A4*	−4.4	0.08	9.8 × 10^−6^	5	4.8 × 10^−2^	0.95	EUR	Height	6.2 × 10^−47^	NA
*BAK1*	−12	0.08	2.8 × 10^−32^	6	2.5 × 10^−3^	1	EUR	Platelet count	2.6 × 10^−149^	0.97
*GPR84*	−5.7	0.11	1.4 × 10^−6^	12	3.3 × 10^−3^	1	EUR	Platelet count	3.9 × 10^−47^	NA
*BAK1*	−12	−13	7.0 × 10^−34^	6	3.9 × 10^−3^	1	ALL	Platelet count	2.6 × 10^−149^	0.97
*c6orf1*	7.5	0.74	6.7 × 10^−14^	6	0.21	0.54	ALL	Height	9.0 × 10^−132^	NA
*CETP*	−20	−7.7	4.2 × 10^−73^	16	2.3 × 10^−3^	1	ALL	HDL cholesterol	6.1 × 10^−97^	NA
*NLRC5*	−7.1	−3.7	1.4 × 10^−12^	16	0.31	0.66	ALL	HDL cholesterol	2.0 × 10^−65^	NA
*PGP*	−4.5	−0.04	5.6 × 10^−6^	16	1.3 × 10^−2^	0.95	ALL	Height	1.9 × 10^−32^	NA
*SETD9*	4.6	0.02	4.3 × 10^−6^	5	0.19	0.80	ALL	Height	9.6 × 10^−17^	0.57
*SLC20A2*	−4.5	−0.25	7.9 × 10^−6^	8	0.32	0.68	ALL	MCHC	7.3 × 10^−21^	0.51
*SLC22A4*	−4.7	−0.05	2.4 × 10^−6^	5	0.10	0.89	ALL	Height	6.2 × 10^−47^	NA
*VPS45*	8.8	0.08	1.2 × 10^−18^	1	0.27	0.69	ALL	WBC count	5.8 × 10^−6^	NA
*ZBTB38*	6.7	0.18	2.6 × 10^−11^	3	8.3 × 10^−3^	0.99	ALL	Height	9.5 × 10^−150^	0.58

Details of the studies used in PhenomeXcan are in Table S2.

P3 = COLOC probability eQTL and GWAS signals are independent; P4 = COLOC probability eQTL and GWAS signals are colocalized; AFHI = African American and Hispanic/Latino transcriptome prediction model; EUR = European transcriptome model; ALL = African American, Hispanic/Latino, and European transcriptome model; MESA = Multi-Ethnic Study of Atherosclerosis; PAGE = Population Architecture using Genomics and Epidemiology study; RCP = PhenomeXcan regional colocalization probability.

**Figure 3:**
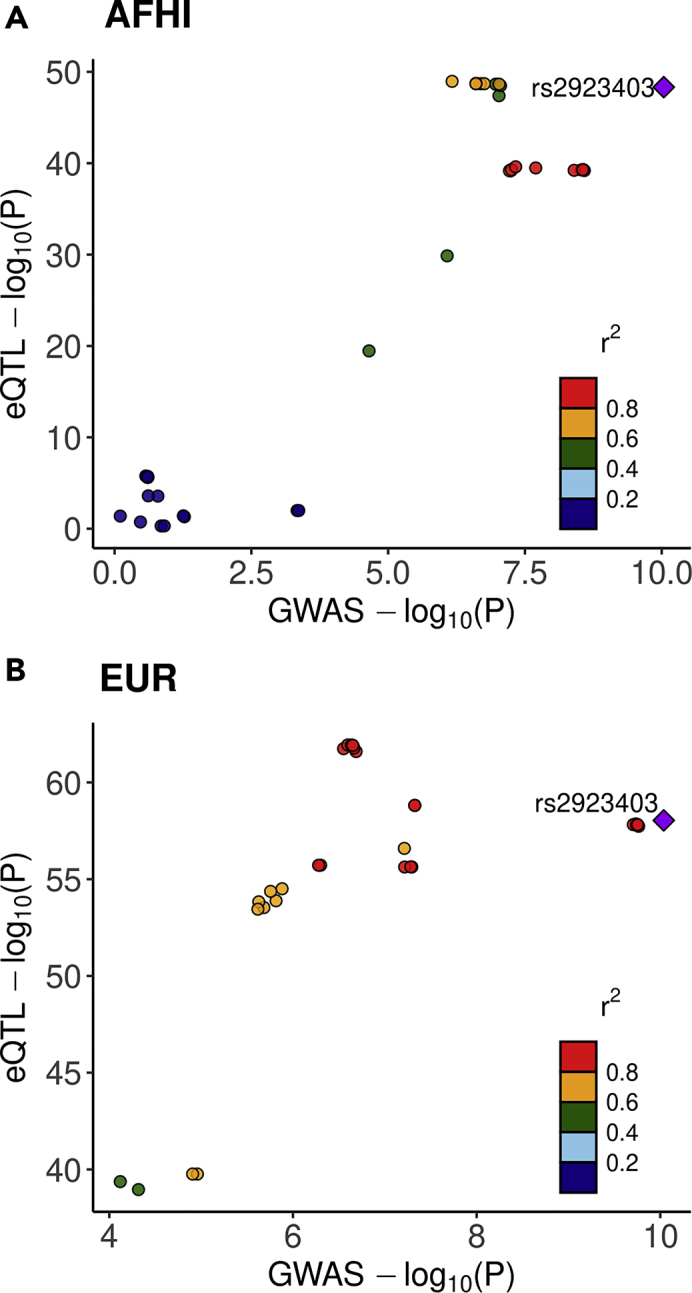
*SMIM19* GWAS and eQTL Signals are Colocalized in AFHI, but not EUR LocusCompare (Liu et al., 2019) plots for mean corpuscular hemoglobin concentration (MCHC) PAGE GWAS p values compared to (A) AFHI MESA eQTL p values and (B) EUR MESA eQTL p values of SNPs in the *SMIM19* prediction models. When most points are located on the diagonal, it indicates the GWAS and eQTL signals are likely colocalized. The lead SNP in the AFHI eQTL and PAGE GWAS, rs2923403, is located among the top signals and in the upper right corner, supporting the COLOC evidence for colocalization AFHI (P4 = 0.90). When using EUR eQTL data in COLOC, the GWAS and eQTL signals did not colocalize (EUR P4 = 0.047). Points are colored according to the pairwise LD r^2^ with rs2923403 in (A) AMR and (B) EUR 1000 Genomes populations.

One gene that was identified as significantly associated with mean corpuscular hemoglobin concentration (MCHC) in both AFHI and EUR at the stringent threshold of 1.1 × 10^−7^ was *SMIM19*. In the PAGE GWAS, SNPs near *SMIM19* were found to be associated with MCHC (Wojcik *et al.*, 2019). In our analysis, *SMIM19* was only found to have colocalized GWAS and eQTL signals with AFHI eQTLs (P4 = 0.90), but not with EUR (P4 = 0.047) or ALL (P4 = 0.052) eQTLs (Figure 3, Table S1). *SMIM19* is also significantly associated with MCHC (P = 2.81 × 10^−23^, RCP = 0.578) in PhenomeXcan with GWAS summary statistics from the UKBioBank. A gene located next to *SMIM19* on chromosome 8, *SLC20A2*, associated with MCHC and had colocalized signal with the ALL MESA eQTLs (P4 = 0.68). *SLC20A2* is also significantly associated with MCHC (P = 7.28 × 10^−21^, RCP = 0.507) in PhenomeXcan with GWAS summary statistics from the UK BioBank. While both genes may be involved in MCHC, in our study, *SMIM19* has stronger evidence of acting through gene expression regulation to affect MCHC than *SLC20A2* as indicated by higher P4 in PAGE using AFHI, higher cross-validated prediction performance in all populations, and higher RCP in PhenomeXcan (Tables S1 and S2).

Of the 17 unique gene-trait pairs that replicated in PhenomeXcan, 5 of these gene-trait pairs do not appear in the GWAS Catalog and thus may represent new biology discovered through TWAS. These include *ISCA2*, *SETD9*, and *SLC22A4*, associated with height; *VPS45* associated with WBC count; and *GPR84* associated with platelet count. *ISCA2, SETD9*, *SLC22A4*, and *VPS45* were significant in AFHI S-PrediXcan while only *SLC22A4* and *GPR84* were significant in EUR S-PrediXcan. *SETD9*, *SLC22A4*, and *VPS45* were significant in ALL S-PrediXcan.

The other 12 gene-trait pairs that replicated in PhenomeXcan were found significant in at least one other GWAS of the same or similar phenotype. In the original PAGE GWAS, *BAK1* in relation to platelet count, *CETP* in relation to HDL cholesterol, *c6orf1* in relation to height, *ZBTB38* in relation to height, and *SMIM19* in relation to MCHC were all mapped as genes nearest to the significantly associated SNP (Table S3).

## Discussion

We applied S-PrediXcan to GWAS results of 28 traits from the PAGE study and found a higher proportion of genes with colocalized GWAS and eQTL signals that replicated in PhenomeXcan using the AFHI transcriptome models than with using EUR or ALL models. This suggests that through using population-matched gene expression prediction models, we find more significant gene-trait pairs that replicate in larger, independent studies. We found that S-PrediXcan Z-scores are consistent between AFHI and EUR transcriptome models (R = 0.63), even if a particular gene was only found significant using one or the other population (Figure 1). As has been shown in SNP effect size comparisons (Stranger et al., 2012; Marigorta and Navarro, 2013; Wojcik *et al.*, 2019; Shang *et al.*, 2020), this strong gene effect size correlation indicates the underlying biological pathways affecting each complex trait do not differ between populations. Instead, our power to detect the associations differs and subsequently, predictive power between populations is reduced (Mogil et al., 2018; Martin et al., 2019; Keys et al., 2020). We have more power to detect associations in PAGE that replicate in independent cohorts using the AFHI transcriptome prediction model because the minor allele frequency and LD structure of AFHI more closely resembles that of PAGE than does the structure of either EUR or ALL (Mogil *et al.*, 2018; Wojcik *et al.*, 2019).

Four gene-trait pairs that replicated in PhenomeXcan mapped as the nearest gene to an associated SNP locus in the original PAGE study (Wojcik *et al.*, 2019). These include *BAK1*, where here we found increased predicted *BAK1* associated with decreased platelet count using all three transcriptome models. We identified *CETP* using the ALL and AFHI models, *SMIM19* using the AFHI transcriptome model, and *ZBTB38* using the EUR and ALL transcriptome models. Increased predicted *CETP* associated with decreased HDL cholesterol levels, supporting previous findings (Barter *et al.*, 2003; Thompson et al., 2003; de Grooth *et al.*, 2004; Kosmas *et al.*, 2016; Andaleon et al., 2019). Increased predicted *SMIM19* expression associated with decreased MCHC. In addition to associating in the original PAGE GWAS, SNPs near *SMIM19* associated with MCHC in two independent GWAS (Hodonsky *et al.*, 2017; Astle *et al.*, 2016). Meanwhile, we found increased predicted *ZBTB38* expression associated with increased height. This association is supported by 17 other independent GWAS (Gudbjartsson *et al.*, 2008; Lettre *et al.*, 2008; Sanna *et al.*, 2008; Weedon *et al.*, 2008; Cho *et al.*, 2009; Soranzo *et al.*, 2009; Kamatani *et al.*, 2010; Kim *et al.*, 2010; Lango Allen et al., 2010; N'Diaye *et al.*, 2011; Bernt *et al.*, 2013; Wood *et al.*, 2014; He *et al.*, 2015; Nagy *et al.*, 2017; Tachmazidou *et al.*, 2017; Kichaev, 2018; Akiyama *et al.*, 2019; Wojcik *et al.*, 2019).

Although not identified in the original PAGE GWAS (Wojcik *et al.*, 2019), SNPs near *PGP* associated with height in European and Japanese GWASs (Tachmazidou *et al.*, 2017; Akiyama *et al.*, 2019). We found increased *PGP* predicted expression associated with decreased height, thus providing more evidence *PGP* affects height through gene expression regulation. Similar to *PGP*, *SLC20A2* was not identified in the original PAGE GWAS but replicated in PhenomeXcan. We found SNPs near *SLC20A2* associated with MCHC in independent GWAS (Kanai *et al.*, 2018), and SNPs near *SLC20A2* were also associated with mean corpuscular hemoglobin volume, a related phenotype to MCHC, in three other independent GWAS (Astle *et al.*, 2016; Kanai *et al.*, 2018; Chen *et al.*, 2020). Here, we found increased *SLC20A2* predicted expression associated with decreased MCHC. More work is needed to disentangle whether *SMIM19* or *SLC20A2,* which are located next to each other on chromosome 8, is causal for MCHC. In our study, *SMIM19* has stronger evidence of acting through gene expression regulation to affect MCHC, but both genes may be involved.

We discovered several gene-trait associations that replicated in PhenomeXcan but were not previously included in the GWAS Catalog and thus may represent new biological mechanisms underlying the traits. These include *ISCA2*, *SETD9*, *SLC22A4*, *VPS45*, and *GPR84.* Neither *ISCA2* nor *SETD9* were previously identified in GWAS as associated with height; we found increased expression of these genes associated with increased height. *SLC22A4* was not previously identified as associated with height despite our findings demonstrating increased *SLC22A4* expression is associated with decreased height. Similarly, no previous GWAS have linked increased *GPR84* expression to increased platelet count. Mutations in *VPS45* are known to cause neutrophil defect syndrome (Vilboux *et al.*, 2013; Stepensky *et al.*, 2013), and we found significant associations between predicted *VPS45* expression and WBC count.

There are significantly more genes with no evidence of colocalization nor evidence of independence when analyzing the AFHI S-PrediXcan output. These 50 genes could be functioning through gene expression regulation. Better methods, specifically colocalization methods for recently admixed populations, are needed to determine whether these genes are likely functional.

In summary, we found more gene-trait pairs discovered in PAGE with AFHI transcriptome models replicated in PhenomeXcan (11/32, 34%) compared to the gene-trait pairs discovered with EUR models (5/20, 25%) and, to a smaller extent, ALL models (10/37, 27%). Since the largest populations in PAGE are of Hispanic/Latino and African American ancestries, TWAS with population-matched transcriptome models, i.e. AFHI rather than EUR, have more power for discovery and discovered genes are more likely to replicate. Transcriptome prediction models trained in a cohort with similar ancestries to the original GWAS should be used and thus more transcriptome studies in diverse populations are needed.

### Limitations of the Study

Here we identified gene-trait pairs using MESA transcriptome models in conjunction with the PAGE GWAS summary statistics in a TWAS analysis. The MESA models were trained using monocyte transcriptomes, and other tissues are likely more relevant to the phenotypes studied. Better complex trait methods for handling linkage disequilibrium and local ancestry in admixed populations like PAGE and MESA are needed. While the GWAS summary statistics from the combined PAGE populations are currently available in the GWAS Catalog, making within population summary statistics publicly available in future studies will encourage meta-analyses and promote development of more sophisticated models to help narrow the diversity gap in genomics (Peterson *et al.*, 2019; Ben-Eghan *et al.*, 2020). More genomes and transcriptomes in more tissues in admixed populations are needed to enhance model development and to better understand the genetics of complex traits in all populations.

### Resource Availability

#### Lead Contact

Further information and questions should be directed to and will be fulfilled by the Lead Contact, Heather Wheeler (hwheeler1@luc.edu).

#### Materials Availability

This study did not generate new unique reagents.

#### Data and Code Availability

All scripts used for analyses are available at https://github.com/WheelerLab/MESA_expression_prediction. TWAS summary statistics, colocalization results, and MESA models from this study can be found at Mendeley Data: https://doi.org/10.17632/p8cgvyz4sz. PAGE GWAS summary statistics are available in the GWAS Catalog at https://www.ebi.ac.uk/gwas/publications/31217584.

## Methods

All methods can be found in the accompanying Transparent Methods supplemental file.

## Supplemental Information

Document S1. Transparent Methods, Figure S1, and Table S3

Table S1. PAGE S-PrediXcan Results and COLOC Results, Related to Table 2

Table S2. PhenomeXcan Replicated Gene-Trait Pairs, Related to Table 2
